# Case Report: First case of synchronous androgen receptor-positive salivary duct carcinoma and prostate adenocarcinoma

**DOI:** 10.3389/fsurg.2026.1697475

**Published:** 2026-03-26

**Authors:** Sofia Kourou, Louis Jansen, Lisa Nachtsheim, Julia van de Loo, Christoph Arolt, Lena Hieggelke, Alexander Quaas, Jens Peter Klussmann, Marcel Mayer

**Affiliations:** 1 Department of Otorhinolaryngology, Head and Neck Surgery, Medical Faculty, University of Cologne, Cologne, Germany; 2 Institute of Pathology, Medical Faculty, Medical Faculty, University of Cologne, Cologne, Germany

**Keywords:** androgen receptor, head and neck carcinoma, metastasis, prostate carcinoma, salivary duct carcinoma, targeted therapy

## Abstract

**Introduction:**

The synchronous occurrence of two androgen receptor-positive (AR+) malignancies with overlapping metastatic patterns is exceedingly rare and poses significant diagnostic and therapeutic challenges. Salivary duct carcinoma (SDC) is an aggressive AR + tumor with a high propensity for early metastasis, whereas low-risk prostate adenocarcinomas (PCa) are often indolent. We report the first case of a synchronous SDC of the right parotid gland and a low-grade PCa, where the origin of osseous metastatic spread was initially unknown.

**Case presentation:**

A 65-year-old male patient presented with progressive right-sided facial palsy and multiple osseous lesions. Initial histopathological assessment of the parotid mass and assignment of the metastases remained inconclusive. After performing a prostate-specific membrane antigen-positron emission tomography/computed tomography (PSMA-PET-CT) scan, a low-grade prostate adenocarcinoma (PCa) (Gleason 3 + 3) was diagnosed. Further pathological and imaging evaluation revealed an AR+, HER2-low, CK7-positive SDC. The extensive skeletal metastases were attributed to the SDC.

**Conclusion:**

This case shows the diagnostic complexity of synchronous AR + malignancies with overlapping metastatic profiles and emphasizes the need for integrated clinical, radiological, and pathological assessment to ensure optimal patient management. It highlights the need to biopsy metastatic bone lesions in cases of SDC—especially in older men—to rule out osseous metastases from more frequent carcinomas such as PCa.

## Introduction

The simultaneous manifestation of two distinct but molecularly similar malignancies presents an exceptional challenge for modern oncology. The situation becomes particularly complex when both tumors exhibit overlapping metastatic patterns, imaging characteristics and molecular profiles, such as androgen receptor (AR) expression. In such cases, clinical presentation, radiological findings, and even pathological diagnostics can lead to considerable uncertainty regarding the precise attribution of metastases, with direct implications for therapeutic planning and patient survival.

Salivary duct carcinoma (SDC) is a rare, high-grade malignancy predominantly found in the major salivary glands, accounting for up to 18% of all salivary gland carcinomas, and primarily affecting men over the age of sixty (1). SDC is characterized by aggressive biology with rapid local progression, early perineural invasion, and a pronounced tendency for lymphatic and hematogenous metastasis, especially to the bones and the lung. Histologically, SDC resembles ductal breast carcinoma and frequently displays strong AR expression and, in a subset of cases, HER2 overexpression (2, 3). The overall prognosis is poor, with five-year disease-free survival rates below 50% and a median survival of usually less than three years. Therapeutic options are limited: while localized tumors are treated surgically and with adjuvant radiation therapy, metastatic disease is managed with targeted therapies against AR, HER2, or platinum-based chemotherapy (4–6).

Prostate adenocarcinoma (PCa) is the most common malignancy in men and is characterized by a strong dependence on AR signaling (7). While high-risk and advanced PCa frequently metastasizes to the bone, low-risk PCa is typically indolent and rarely associated with distant metastases (8).

When two AR-positive (AR+) malignancies occur simultaneously and both have the potential to metastasize to the skeleton, significant diagnostic uncertainty arises. The overlap of clinical, radiological, and immunohistochemical features may lead to misdiagnosis, delays in therapy initiation, and, in the worst case, inadequate treatment.

We present the first case of a patient with synchronous AR + SDC of the right parotid gland and a low-risk PCa, both diagnosed in the context of extensive bone metastases. The report highlights the need to take biopsies from metastatic bone lesions in cases of SDC—especially in older men—to rule out osseous metastases from more frequent carcinomas such as PCa.

## Case presentation

A 65-year-old male patient first noticed progressive right-sided facial palsy in June 2024. He presented to both Departments of Neurology and Otolaryngology—Head and Neck Surgery. Initially, oral corticosteroid therapy was started, under which the palsy progressed. Several further neurological evaluations and imaging studies were performed. The first magnetic resonance imaging (MRI) of the neck and computed tomography (CT) of the neck in November and December 2024 revealed a mass in the right parotid gland with suspected multiple osseous metastases in the spine and in the acetabulum. Based on these findings, the patient was referred to another Department of Otolaryngology—Head and Neck Surgery where a core needle biopsy of the parotid gland lesion was performed in December 2024. Initial external pathology described a “spindle cell neoplastic process” with the following features: spindle cell proliferation, partially perineural carcinoma infiltrates, Ki-67 around 30%, disseminated pan-cytokeratin, AE1/3 and CK7 positive tumor cell infiltrates. The markers p40, TTF1, GATA3, CK20, NKX3.1, PSMA, Pax8, synaptophysin, CDX2, S100, CD31, and ERG were negative. The exact tumor entity remained unclear.

For further clarification, an open biopsy of the parotid gland lesion was performed in January 2025. Histopathological assessment showed a malignant tumor with spindle cell component, perineural infiltration, and a Ki-67 of about 30%. The exact tumor entity remained unclear.

**Figure 1:**
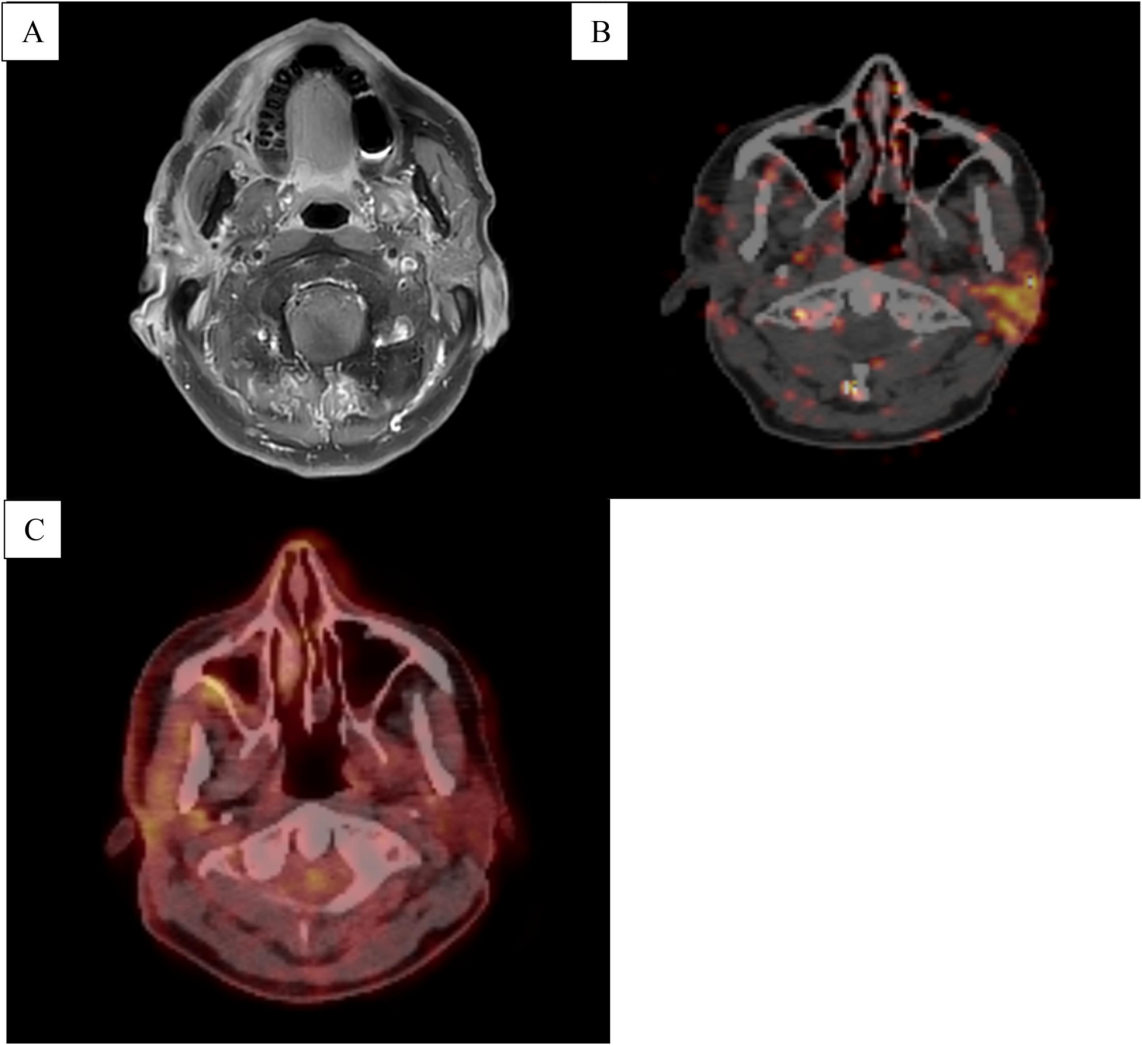
**(A)** T1-weighted MRI scan of the neck with contrast revealing a hyperintense diffuse lesion of the right parotid gland. **(B)** PSMA-PET/CT scan demonstrating tracer uptake in the left parotid gland and a lack of tracer uptake in the right parotid gland suggestive of tumor-destructed glandular tissue. **(C)** FDG-PET/CT scan depicting low-level, focally increased tracer uptake of the right parotid gland.

**Figure 2:**
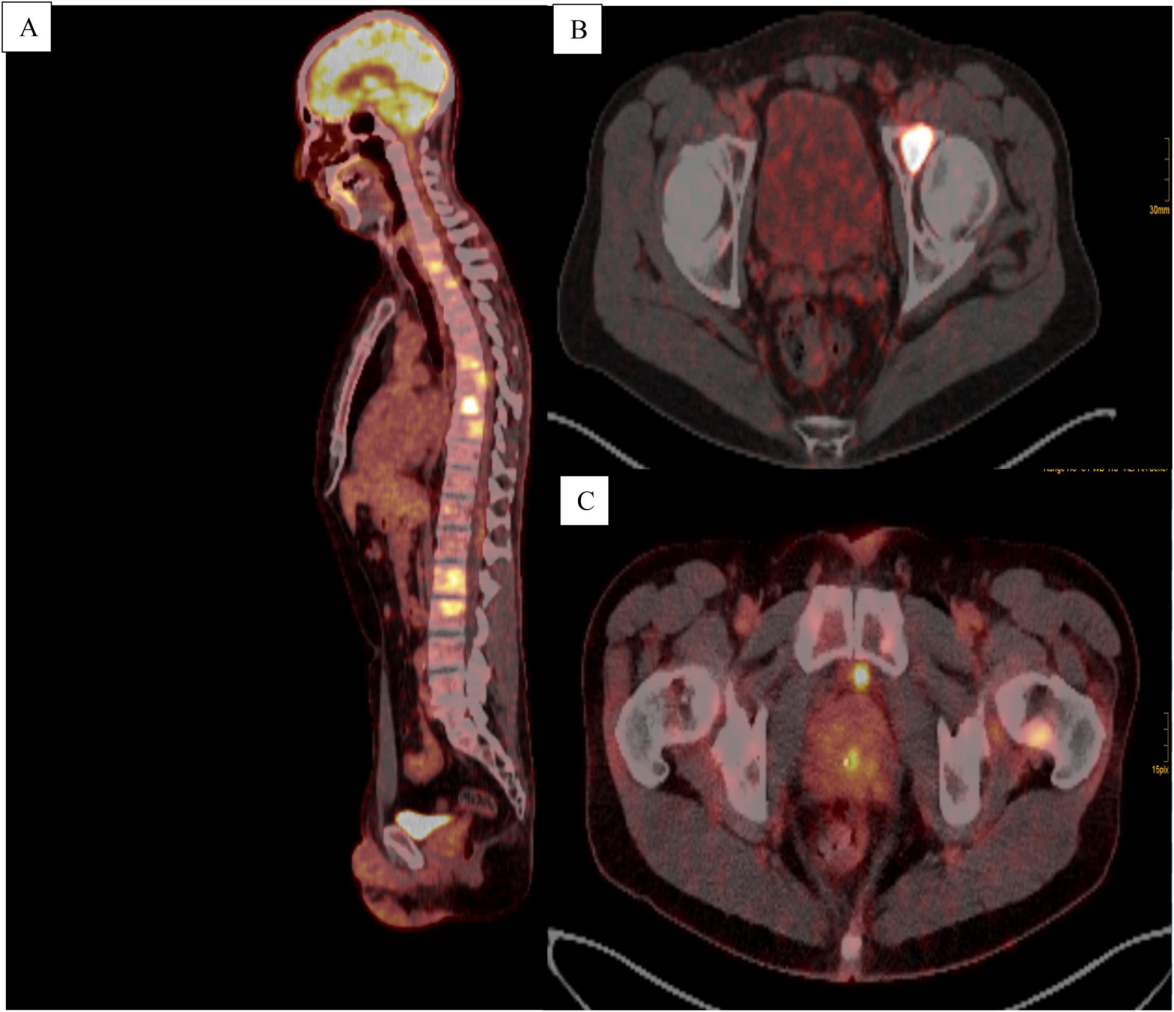
**(A)** Sagittal FDG-PET/CT scan of the trunk and head and neck region illustrating multilocular osseous metastases with increased tracer uptake. **(B)** Axial PSMA-PET/CT scan of the pelvis depicting an osseous metastasis within the left acetabulum. **(C)** Axial FDG-PET/CT scan showing an increased uptake in both the prostate gland and the left acetabulum.

**Figure 3:**
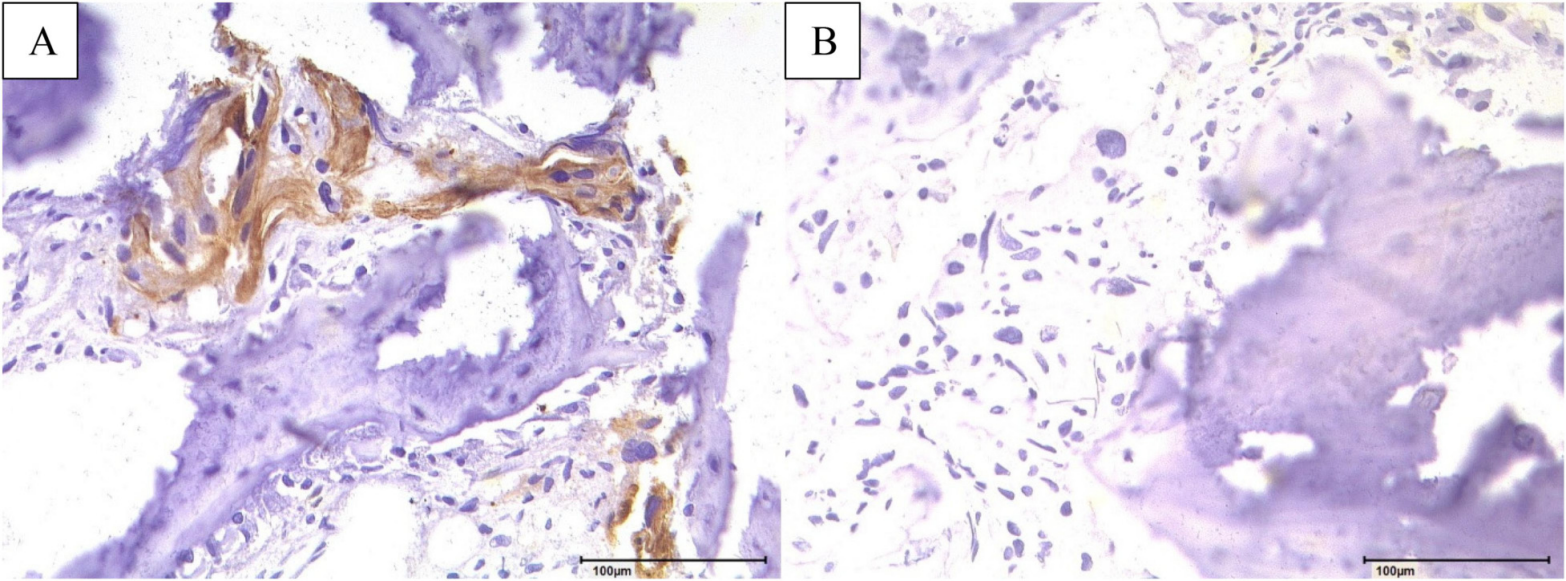
Immunohistochemical analysis of bone metastasis: **(A)** pleomorphic tumor cells with cytoplasmic positivity for CK7 and **(B)** complete nuclear negativity for NKX3.1, 200-fold magnification.

A subsequent fluorodeoxyglucose-positron emission tomography/CT (FDG-PET/CT) scan showed minimal avidity in the right parotid region and intense FDG-positive bone metastases in the spine as well as in the left acetabulum in January 2025 (Figures 1, 2). Additionally, an FDG-avid lesion was detected at the apex of the prostate. Due to these skeletal manifestations, a biopsy of the acetabulum was performed (Figure 3). The initial pathological assessment revealed a poorly differentiated malignant tumor, but the exact origin remained unclear. The patient was referred to our tertiary referral Department of Otolaryngology—Head and Neck Surgery. The external sample from the parotid gland lesion was re-evaluated by a pathologist with special expertise in salivary gland pathology (CA) and a CK7-positive, AR + (85% of tumor cells; moderate to strong) and HER2-low [Immunohistochemistry (IHC) score 1+] SDC was diagnosed. Programmed death-ligand 1 (PD-L1) was negative (TPS 0%, CPS <1%). The case was discussed in the head and neck tumor board. Due to the still unclear primary tumor situation and the FDG-avid lesion at the apex of the prostate, a prostate-specific membrane antigen-positron emission tomography-CT (PSMA-PET-CT) scan as well as a biopsy from the prostate lesion were performed in March 2025 (Figures 1, 2). The PSMA-PET/CT showed an enlarged prostate with heterogeneous, non-focal PSMA positivity, but no definitive lesion. No PSMA-positive lymph node or organ metastases were found but disseminated PSMA-positive osteoblastic lesions were present throughout the skeleton, morphologically appearing typical for PCa metastases. The subsequent prostate biopsy revealed an acinar adenocarcinoma with a Gleason score of 3 + 3 = 6 (WHO grade group 1), with a maximum extent of 0.1 cm (10% of the core) in one of 16 cores. Additionally, tumor markers were determined in the peripheral blood. In February 2025, the prostate-specific antigen (PSA) level was 2.65 µg/L (reference < 3.11 µg/L) and therefore within the normal range. In April 2025, the PSA level increased to 3.66 µg/L, exceeding the upper reference limit. At the same time, free PSA was 0.63 µg/L, resulting in a free PSA/PSA ratio of 0.17 (reference > 0.19). In addition, other tumor markers were assessed. CA 19-9 was 13 kU/L (reference < 34 kU/L), and CEA was 0.8 µg/L (reference < 5.0 µg/L), both within the normal range. The patient's only comorbidities were arterial hypertension and hypercholesterolemia, both of which were well controlled under stable pharmacological management. The case was discussed in an interdisciplinary tumor board. Due to the low Gleason score, low PSA, and lack of aggressive tumor features, the metastases were attributed to the SDC. Active surveillance was recommended for the PCa. The tissue samples from the acetabulum lesion were re-examined to definitively clarify the origin of the osseous metastases in May 2025.

**Figure 4:**
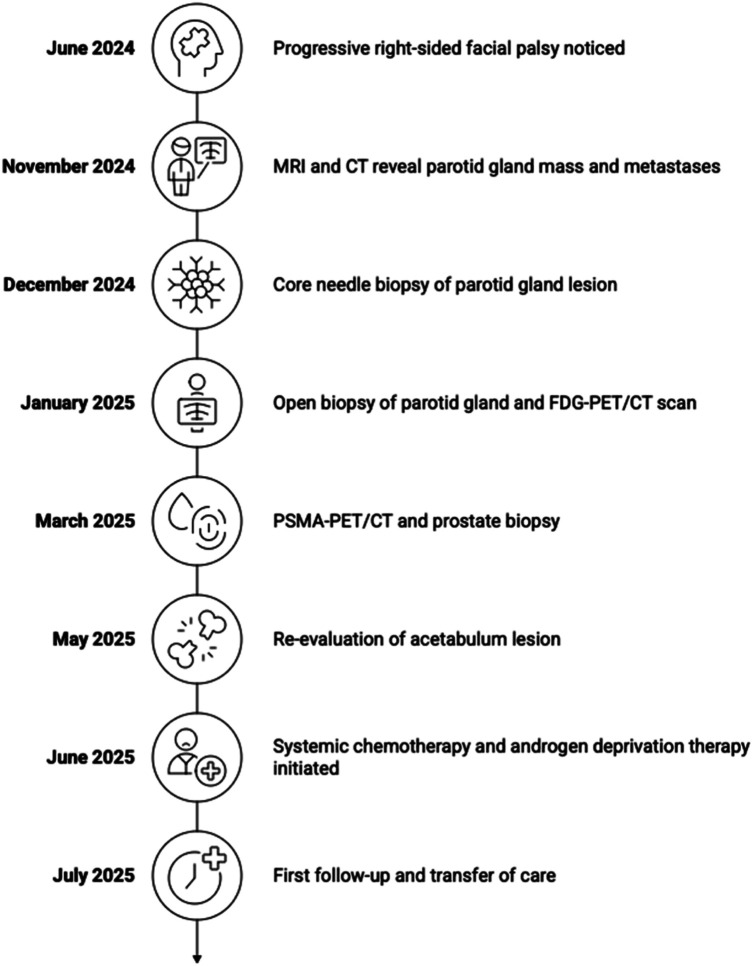
Timeline of the patient's clinical course.

The findings were primarily consistent with a bone metastasis originating from the SDC. Several differential diagnoses were considered and systematically excluded, including bone-metastatic prostate adenocarcinoma, high-grade salivary gland carcinomas with spindle cell features (such as sarcomatoid SDC or myoepithelial carcinoma), and, less commonly, metastatic ductal breast carcinoma or spindle cell squamous cell carcinoma. The combination of strong AR expression, CK7 positivity, HER2-low status, and negativity for NKX3.1, PSMA, S100, and p40 supported SDC and largely excluded prostatic, myoepithelial, and primary squamous cell tumors. Positivity for epithelial markers (AE1/3, CK7) argued against sarcoma or melanoma, while the low-volume Gleason 6 prostate cancer and the integrated assessment of histology, immunohistochemistry, and imaging confirmed that the extensive bone metastases originated from SDC, with the prostate adenocarcinoma considered an incidental synchronous second primary. Systemic chemotherapy with carboplatin and paclitaxel together with a combined androgen deprivation therapy consisting of leuprorelin and bicalutamide was initiated and started in June 2025. Carboplatin (AUC 6) and paclitaxel (200 mg/m^2^) were administered according to a schedule of Day 1 and repeated on Day 21. The planned treatment duration consisted of a total of 6 cycles. Bicalutamide was administered daily at 50 mg, and leuprorelin was given subcutaneously at 3,6 mg once per month. At the first follow-up after one month, the patient reported good tolerability of the therapy (ADT and chemotherapy). He did not report any specific adverse effects. The patient last presented to our department in July 2025. Thereafter, due to the considerable logistical burden associated with repeated travel, systemic therapy (ADT and chemotherapy) was continued at a local oncology center closer to his place of residence. No other follow-up data exist. To provide a clear overview of the clinical course over time, a timeline was developed (Figure 4).

## Discussion

This case illustrates the possible challenges that arise from the synchronous diagnosis of two AR + malignancies — especially when both tumors have the potential to metastasize to the skeleton and show an overlap in imaging and immunohistochemistry. The initial symptom of progressive facial palsy led to a complex, multi-step diagnostic process with repeated neurological, otolaryngological, and radiological evaluations. Diagnosis was significantly complicated due to inconclusive clinical and imaging findings, as well as ambiguous pathological results. A central challenge was the correct attribution of the extensive bone metastases. While the PSMA-PET/CT scan showed disseminated PSMA-positive osteoblastic lesions—a pattern typically seen in metastatic PCa—the low Gleason score, low PSA, and minimal tumor burden in the prostate argued against an aggressive, metastatic disease. Histopathological examination ultimately confirmed that the metastases originated from SDC, thus resolving the diagnostic ambiguity. SDC is known to be the most aggressive entity among all 21 salivary gland carcinomas. A high proportion of patients with SDC experiences locoregional recurrence or distant metastasis within the first two years after diagnosis; median survival is less than three years. Prognosis is particularly poor when facial palsy is present early (4, 9, 10). The rapid growth of the parotid lesion, the swift development of multiple bone metastases, and the progressive facial nerve palsy reflect the high aggressiveness of this tumor type.

A review of the literature shows that there is no report of a synchronous, i.e., simultaneously diagnosed, AR + SDC and PCa. One isolated case report described a patient with a metachronous SDC and a history of PCa, but systematic analyses or larger case series are lacking (11). The present case highlights the critical importance of thoroughly ruling out a synchronous AR + PCa in male patients diagnosed with primary SDC presenting with osseous metastases. This recommendation is grounded in several clinicopathological considerations. First, SDC is a rare but highly aggressive salivary gland malignancy that predominantly affects older men and tends to metastasize early, particularly to the bones, lungs, and liver (10). Second, PCa also shows a high incidence in elderly men and is well known for its strong skeletal tropism (12). Given this epidemiological overlap, a synchronous occurrence of both malignancies — although very rare — should be actively considered in relevant clinical contexts.

A particular diagnostic challenge arises from the fact that both SDC and PCa frequently overexpress AR and prostate-specific markers such as PSA and PSAP (13, 14). In the case presented here, the coexistence of a low PSA level, a low-volume, low-grade PCa (Gleason 6), and widespread PSMA-avid osteoblastic metastases initially led to a diagnostic dilemma. However, the identification of an AR + SDC in the parotid gland, combined with pathological confirmation of SDC in bone lesions, clarified the true source of the metastatic disease. This highlights the necessity of integrating clinical, radiological, and pathological data, including extended immunohistochemical profiling, to accurately differentiate between these two entities. Recognizing the correct tumor origin is essential for appropriate therapeutic decision-making, particularly given the different biological behavior and treatment options available for SDC and PCa. Interpretation of the PSMA-PET/CT scan deserves a particular focus in the present context.

In our case, there was minimal PSMA uptake in the SDC primary tumor, whereas significant uptake was observed in the bone metastases. This observation supports findings from other studies; In a prospective cohort it is reported that only about 40% of SDC patients demonstrated clinically relevant tumor uptake on PSMA-PET (tumor-to-liver ratio > 1), indicating generally low and variable PSMA expression within the tumor tissue (15).

Another study showed immunohistochemically that PSMA expression in SDC is heterogeneous. It demonstrated that the majority of samples in a small cohort were PSMA-negative (16). Taken together, these data suggest that PSMA-PET imaging of SDC is highly heterogeneous, with only a minority of cases exhibiting clinically significant uptake.

A particular focus should be placed on the increased PSMA uptake observed in the healthy left parotid gland in our case. This finding is consistent with existing literature demonstrating that healthy salivary glands physiologically exhibit very high and reproducible PSMA ligand uptake on PSMA-PET imaging, especially in the major salivary glands such as the parotid gland (17). This uptake reflects the normal expression of PSMA in the ductal and acinar cells of the glandular tissue (18). In our case, PSMA-PET/CT was therefore not a reliable criterion for assigning the bone metastases but had to be supplemented by comprehensive immunohistochemical and clinical evaluation.

In cases of synchronous AR + PCa and SDC, androgen deprivation therapy (ADT) emerges as a particularly relevant therapeutic option, as both tumors are potentially androgen-responsive. However, the duration of response differs substantially between the two entities: while PCa can show a durable response to ADT with a median progression-free survival (mPFS) up to 49 months, the response in SDC is typically markedly shorter with reported mPFS of lower than 9 months (19, 20).

This highlights the need to consider additional targeted treatment strategies for SDC. In HER2-positive SDC, the antibody-drug conjugate trastuzumab-deruxtecan has recently shown promising results, with pooled data from phase I studies reporting mPFS up to 20 months. These findings suggest that HER2 testing should be routinely performed in SDC to identify candidates for HER2-targeted therapies (21).

A key consideration in such cases is the timing of the diagnostic work-up. The need to exclude a synchronous prostate carcinoma must be weighed against the urgent initiation of systemic therapy for aggressive SDC. A parallel diagnostic approach (including simultaneous imaging, PSA testing, and histological assessment of the SDC) should allow for rapid differentiation. If uncertainties persist, the aggressiveness of SDC justifies prioritizing its treatment, with ADT providing interim coverage for both malignancies, while further diagnostics can subsequently continue in parallel.

Despite the clinical insights provided, several limitations of this case must be acknowledged. Most importantly, follow-up data are limited, as the patient continued most of his systemic treatment and subsequent surveillance at a local center close to his home. Consequently, detailed longitudinal data regarding treatment response, radiologic evolution, side effects, and long-term clinical outcome were not available to us. The duration of follow-up is therefore restricted, and robust assessment of therapeutic efficacy and tolerability is not possible. In addition, this report describes a single patient. As an isolated case report, it does not allow for systematic comparison with additional cases or for drawing broader conclusions regarding diagnostic algorithms or therapeutic strategies. The rarity of synchronous AR-positive SDC and PCa further limits external validation. These limitations underline that the observations presented here should be interpreted cautiously and primarily serve to raise awareness of a rare but clinically relevant constellation.

In summary, this case demonstrates that in synchronous AR + malignancies, imaging-based diagnostics alone are insufficient. The integration of clinical, imaging, and especially pathological findings with a broad immunohistochemical panel is essential. Given the aggressive biology of SDC, rapid and targeted therapy planning is crucial to avoid further deterioration of prognosis.

## Conclusion

We report the first case of a synchronous AR + SDC with extensive osseous metastasis and a low-risk PCa. The case underscores the importance of excluding a synchronous AR + adenocarcinoma of the prostate in case of a primary SDC with osseous metastases as patient characteristics of both malignancies overlap. Androgen-deprivation therapy has relevance in case of two synchronous AR + malignancies but has been shown to have limited duration of response in SDC compared to PCa. Therefore, further research is needed to optimize systemic therapy for recurrent and/ or metastatic SDC and to refine imaging strategies in patients with multiple primary tumors.

## Patient perspective

The patient presented for a second opinion from another clinic due to unclear findings regarding the tumor entity. This uncertainty caused some distress and anxiety, as a definitive diagnosis had not yet been established.

## Data Availability

The raw data supporting the conclusions of this article will be made available by the authors, without undue reservation.
